# Rapid analgesia for prehospital hip disruption (RAPID): protocol for feasibility study of randomised controlled trial

**DOI:** 10.1186/s40814-016-0115-6

**Published:** 2017-01-23

**Authors:** Jenna K. Bulger, Alan Brown, Bridie A. Evans, Greg Fegan, Simon Ford, Katy Guy, Sian Jones, Leigh Keen, Ashrafunnesa Khanom, Ian Pallister, Nigel Rees, Ian T. Russell, Anne C. Seagrove, Helen A. Snooks

**Affiliations:** 1 Swansea University Medical School, ILS2, Singleton Campus, Swansea University, SA2 8PP Swansea, UK; 2 Patient Representatives, Swansea, UK; 3 Abertawe Bro Morgannwg University Health Board, Swansea, UK; 4 Welsh Ambulance Services NHS Trust, Swansea, UK

**Keywords:** Paramedic, Hip fracture, Fractured neck of femur, Analgesia, Prehospital, Pain

## Abstract

**Background:**

Adequate pain relief at the point of injury and during transport to hospital is a major challenge in all acute traumas, especially for those with hip fractures, whose injuries are difficult to immobilise and whose long-term outcomes may be adversely affected by administration of opiate analgesics. Fascia iliaca compartment block (FICB) is a procedure routinely undertaken by doctors and nurses in the emergency department for patients with hip fracture but not yet evaluated for use by paramedics at the scene of emergency calls.

In this feasibility study, we aim to test whether FICB administered by paramedics at the scene of participants’ hip fractures is feasible, safe and acceptable. This will enable us to decide whether to proceed to a fully powered, multi-centre pragmatic randomised trial to evaluate whether the procedure is effective for patients and worthwhile for the NHS.

**Methods/design:**

In this study, we propose to recruit ten paramedics in an urban area of South Wales. We will train them to carry out FICB when they attend patients with hip fracture. We will randomly allocate eligible patients to FICB or usual care using audited scratch cards. We will follow up participants to assess measurability of key outcomes including quality of life, pain scores, adverse events, length of stay in hospital, acceptability to patients and compliance of paramedics. We will assess whether the findings meet specified feasibility criteria and, if so, plan a full trial.

**Discussion:**

This study will enable us to recommend whether to undertake a definitive trial of FICB by paramedics for hip fracture.

**Trial registration:**

ISRCTN60065373

## Background

It is predicted that 6.3 million hip fractures a year will occur worldwide by 2050 [1]. In the UK, hip fractures result in more admissions to orthopaedic trauma wards than patients with any other injury [2]; this has a huge financial impact on the National Health Service (NHS) [3].

Hip fracture is associated with a high mortality rate (30% at 1 year). Delay to surgery—over 48 h—has a detrimental effect on patient mortality [4–6]. Early surgery has also been shown to reduce the incidence of postoperative pneumonia (relative risk 0.59) and pressure sores (relative risk 0.48) [7]. The National Institute for Health and Care Excellence [8] therefore advises that surgery should take place on the day of admission or the following day.

Prehospital management of patients with hip fracture can cause severe pain as the injury site is difficult to immobilise and ambulance staff must move the patient by lifting and negotiating through obstacles such as stairs and doorways. Paramedics have a range of available pain relief options including paracetamol, opiates and Entonox, with the most frequently administered being intravenous (IV) morphine [9]. However, several studies have suggested that prehospital pain relief for patients with suspected hip fracture is inadequate, with up to 40% of patients not receiving any pain relief at all [10–13]. Following interviews with patients, who believed their prehospital care could be improved, Aronsson et al. [14] recommended that alternative methods of pain relief should be considered before admission to hospital.

Unfortunately, the pharmacokinetics and pharmacodynamics of opiates mean that unpredictable responses can occur, particularly in the elderly; adverse drug effects when morphine is administered are therefore likely and have the potential to delay a patient being taken to theatre [15]. Rainer et al. [16] showed that IV ketorolac was more cost-effective than IV morphine in isolated limb injury; odds of an adverse event with IV morphine were 144 times (95% confidence interval from 41 to 502) more likely. Alternatives to opiate-based pain relief are therefore desirable in the care of hip fracture.

The prehospital management of hip fractures is vital to provide adequate pain relief, ideally without potential side effects that may delay surgical fixation and have a detrimental effect on patient outcomes. We hypothesise that the use of fascia iliaca compartment block (FICB) to provide pain relief to patients with a broken hip before they are taken to the emergency department (ED) may improve the outcome for patients with hip fracture in this way.

FICB is increasingly used in ED and orthopaedic wards in the care of patients with hip fracture. The technique was first described by Dalens et al. in 1989 [17]. Lees et al. [18] demonstrated statistically significantly reduced pain scores in patients receiving FICB in hospital, compared with controls. Lengths of stay and mortality rates were also reduced, although there may have been confounding variables [18]. Increasingly, the evidence in the literature suggests that FICB is easy to learn, provides better pain relief and triggers fewer side effects than opiates [19–26].

The Association of Anaesthetists of Great Britain and Ireland supports FICB delivered by non-physician practitioners when a physician is not immediately available [27]. The Association states that non-medical registered health professionals can perform FICB if they have been appropriately trained and are following agreed clinical governance procedures.

### Feasibility study aim

The aim of the study is to assess the feasibility of undertaking a fully powered, multi-centre pragmatic randomised trial to test the clinical and cost-effectiveness of paramedics providing FICB as early pain relief for patients who have fractured a hip at the scene of their injury.

### Feasibility study objectives

To assess:

1. Accuracy of recognition of hip fracture by paramedics and thus the safety and feasibility of FICB
2. Willingness of both patients and paramedics to participate in the study
3. Compliance with the FICB protocol by paramedics
4. Sample size required for a full randomised controlled trial (RCT) and recruitment period required to achieve this target
5. Acceptability of FICB as method of providing pain relief in prehospital care of patients with hip fracture
6. Which outcome measures to use in a full RCT and at what point: for example, pain scores before and after pain relief; whether the administration of FICB in prehospital care yields benefits for patients besides pain relief, notably side effects of opioids (nausea, constipation, respiratory depression and confusion); length of time before surgery; and length of stay in hospital. We shall also assess the ability of participants to complete forms, the incidence of missing data and the time taken to complete data collection
7. Whether study processes and outcomes achieve specified feasibility criteria for trial implementation

## Methods/design

### Design

Single-centre randomised parallel-group feasibility trial.

### Setting

The scenes of patients’ injuries, in the predominantly urban catchment area of one ED in South Wales, where the average job cycle time is 90 min.

### Paramedic participants

We have recruited ten paramedics based at ambulance stations in that catchment area and trained them in FICB.

### Inclusion criteria

Adult patients (aged 18 years or older) who are:

- Attended out of hospital by a participating emergency paramedic following a 999 call
- Assessed by the attending paramedic as having an isolated hip fracture; conscious (Glasgow Coma Scale score at least 13) and haemodynamically stable
- Conveyed to the participating hospital

We exclude patients if they refuse analgesia or if the emergency paramedic is working alone without back-up from the advanced paramedic practitioner, emergency medical technician or other paramedic.

### Sample size

In this randomised feasibility study, we are not seeking to evaluate FICB and have not therefore undertaken a formal power calculation. However, considering that the participating hospital treated about 370 patients with hip fracture over 12 months in 2013–2014, and accounting for shift patterns of paramedics and the number of patients excluded for other reasons, we estimate that ten trained paramedics can recruit 50 eligible patients into the trial over 12 months—enough to assess whether the trial meets our specified feasibility criteria for progressing to a full trial.

## Intervention

### Paramedic training

Our proposed training reflects published methods for training non-medical healthcare professionals [28, 29] and expert advice from consultants in anaesthetics and trauma surgery who have previously trained nurses to carry out FICB in the ED. We train paramedics through an online package including a video showing the administration of FICB, followed by group sessions led by a consultant anaesthetist (SF). Pairs of paramedics then attend sessions at the participating hospital where they administer FICB to real patients observed by an anaesthetist. They alternate between administering and critiquing the FICB to ensure their learning is active [30]. The paramedics must pass a competency assessment before they can recruit patients to the study.

The paramedics will attend a 6-month refresher halfway through the year of patient recruitment. We advise them to contact the paramedic research support officer if they feel they need additional training or support during the recruitment period, typically from the consultant anaesthetist.

Our paramedics routinely measure pain scores on a numerical rating scale, a standard tool used by healthcare professionals. The patient clinical record used by paramedics already has a designated box to collect these data as a standard part of the patient’s assessment. We therefore provide no additional training for recording pain scores within this trial.

### Treatment protocol

We give paramedics a printed treatment protocol as an aide mémoire during recruitment. Should a patient be randomly allocated to receive FICB, the paramedic will ensure the participant has no contraindication to FICB including allergy to local anaesthetic; use of anticoagulants; neurovascular damage to the affected leg; previous femoral bypass surgery; infection at the site of injection; inability to palpate the femoral artery on the affected leg; hip prosthesis on the affected side; pregnancy; or body mass apparently less than 50 kg.

### Usual care

At present, paramedics provide a range of analgesia to patients with hip fracture, including IV morphine, paracetamol and Entonox [10]. Participants allocated at random to the control group receive this usual pain relief, as judged appropriate by the paramedic. To participants in the intervention group, paramedics can offer paracetamol and Entonox in addition to FICB. We advise paramedics not to give morphine for at least 20 min after patients have received FICB. If FICB has not relieved the pain after 20 min, however, they can give morphine if they judge that appropriate.

### Randomisation

Before recruiting participants, we produced 100 sequentially numbered scratch cards with concealed trial allocations generated by the trial statistician (GF). To avoid subversion of the randomisation procedure, in particular tampering with the scratch cards, we check that study paramedics use these scratch cards for eligible patients in sequence and account for them on their ambulance stations’ randomisation log. We regularly audit these logs against the remaining scratch cards.

### Patient consent

We take consent in two stages:

1. *Consent to treatment*: paramedics obtain oral consent to treatment (i.e. analgesia) according to usual practise, for example for cannulation or venepuncture.
2. *Consent to participation in the trial*: An NHS researcher seeks retrospective consent from patients to take part in the trial within ten working days of their injury. This usually occurs in hospital, or in the community if necessary. For patients with cognitive impairment, we seek consent to take part in the trial from relatives or carers. Thus, we recognise that it is not ethically appropriate to consent patients to research in a medical emergency [31]. Indeed, in our SAFER randomised trials in emergency care [32, 33], we gained ethical information governance and research approvals to inform patients of their potential inclusion in research within 10 days of attendance by emergency ambulance. The PARAMEDIC trial used similar of enrolling patients, using a waiver of consent under the Mental Capacity Act, and asking patients for consent to follow up after this [34]. Consistent with those trials, all patients who dissent from RAPID then leave the study.

### Study flowchart

**Fig. 1:**
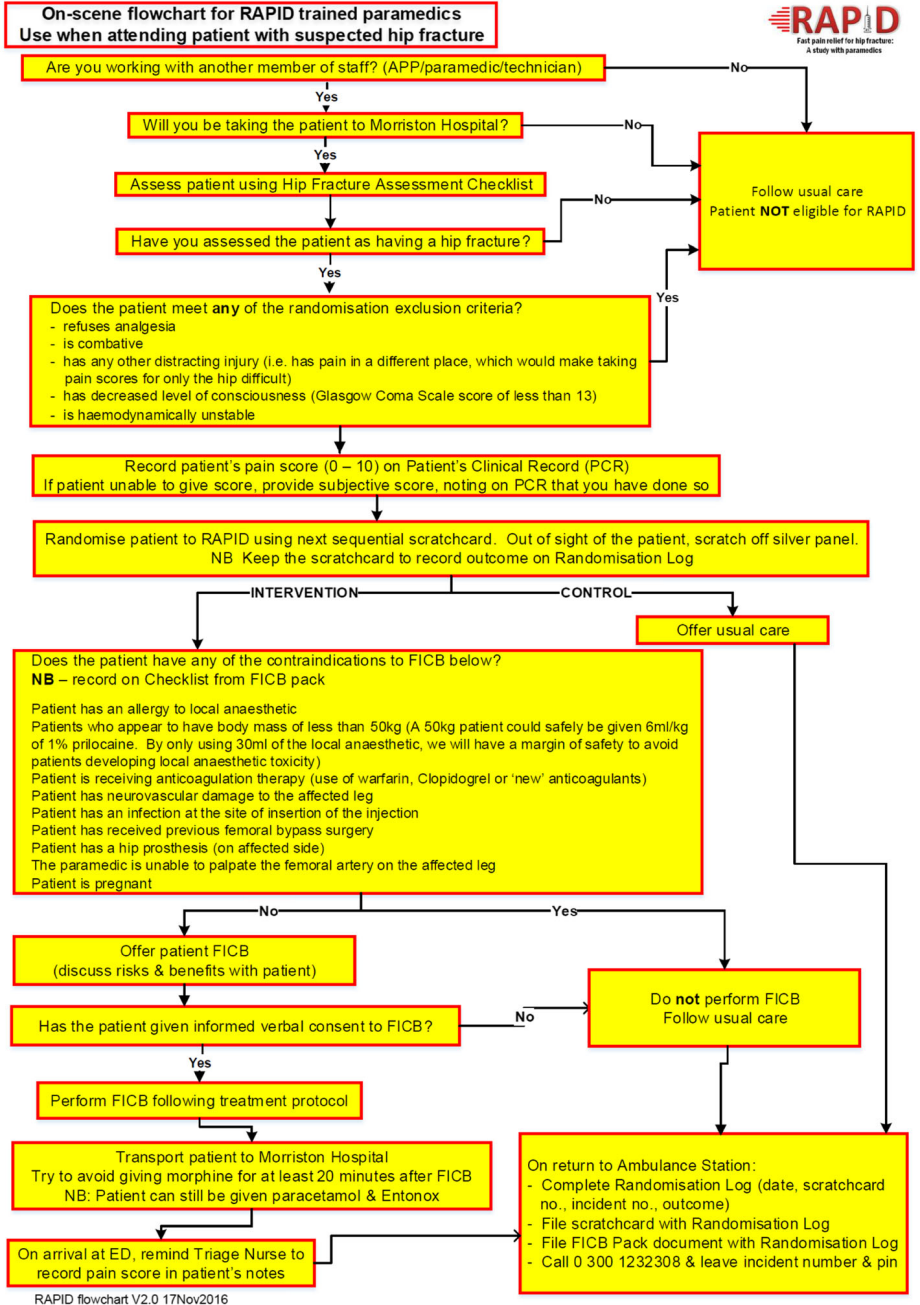
Study flowchart

All study paramedics keep a laminated copy of the study flowchart as an aide mémoire (Fig. 1).

### Outcomes

We are testing the following outcome measures for the full RCT:

#### Primary

Self-reported:

- Health-related quality of life, using SF-12 [35] at 30 days and 6 months

Routinely collected:

- Mortality at 6 months

#### Secondary

Self-reported:

- Mobility score, using the Rivermead Mobility Index at 30 days and 6 months [36]
- Initial pain score at the scene of participants’ injuries before pain relief, using an 11-point numeric rating scale with 0 showing no pain at all and 10 showing the worst pain imaginable [37]
- Satisfaction with care received from paramedics using a modified Quality of Care Monitor [38] 30 days after the participant’s injury

Routinely collected:

- Length of inpatient stay
- Pain score on arrival in the ED using the same 11-point numeric rating scale [37]
- Participant safety assessed by adverse events (AEs) occurring in each trial arm, notably their severity, causality and expectedness
- Duration of paramedic’s management of participant (‘job cycle time’)
- Use of anti-emetics and alternative analgesia
- Time between arrival at ED and surgery

### Qualitative data collection

We will interview ten intervention participants to explore their experiences of receiving FICB at the scene of their injury. This will enable us to explore whether this method of providing analgesia for hip fracture is acceptable to patients (Study Objective 5). We will sample respondents purposively [39] to ensure that they cover the range of the following characteristics:

- Age
- Gender
- Previous hip fracture
- Time of injury
- Study paramedic who treated the participant

We shall record and transcribe these interviews.

Towards the end of the recruitment period, we will also conduct paramedic focus groups by inviting all ten trained paramedics to take part. We will explore their experiences of randomising participants and providing FICB at the scene of injury (Study Objectives 2 and 3) and ask how this could work on a larger scale, to aid our planning of a fully powered RCT.

### Data management

We use an electronic data capture and management system designed for clinical trials, to store anonymised data securely [40]. We use participants’ contact details only to arrange interviews and send postal questionnaires and store them on a password-protected computer separate from the trial database. Transcripts of qualitative interviews and focus groups will have participants’ study number but no identifiable information. We shall also store digital recordings on a password-protected computer.

### Analysis

#### Quantitative

Quantitative analysis will enable us to assess our data against the following feasibility criteria [41], which we seek to meet within reasonable limits:

1. Recruit at least ten paramedics to conduct the trial
2. Paramedics recognise hip fracture with sensitivity of 75% and positive predictive value of 85%
3. At least 50% of intervention participants receive the intervention
4. At least 60% of recruited participants consent to follow up
5. Retrieve primary outcomes for at least 70% of consented participants
6. Mean participant satisfaction in intervention group is at least 80% of that in control group
7. Clinicians are in equipoise about safety and effectiveness of paramedic-administered FICB
8. Balance of serious AEs between groups

We base the criteria for progression on direct measures of a count (criterion 1) and direct measures of proportions (criteria 3–6). We will use receiver operating characteristic analysis to generate the positive predictive value (criterion 2) with the first X-ray providing the gold standard.

To judge whether criterion 7 has been met, we shall compare health-related quality of life, mobility, satisfaction with care and change in pain scores following analgesia using *t* tests of mean scores, mortality risk ratios, median lengths of inpatient stay, paramedic job cycle time and time between arrival at ED and surgery using a Wilcoxon non-parametric test. These exploratory analyses will also enable us to estimate the sample size for a full trial.

We shall report the results of this feasibility trial according to the CONSORT guidelines [42].

#### Qualitative

We will conduct thematic analysis of data from interviews and focus groups. This is a systematic and transparent method of analysis which generates themes from the implicit and explicit ideas contained in the original accounts of participants. One researcher will lead the analysis. Two others, including a lay representative, will independently monitor key stages of coding, generating themes and interpretation and adopt a critical stance to test and confirm findings [43–45].

### Lay contribution

Lay representatives with experience and knowledge of hip fractures and emergency care contributed to developing the research plan, drafting the application for funding and preparing all documentation, notably the Patient Information Sheet. Two lay representatives will sit on the Trial Management Group and two others on the Trial Steering Committee throughout the trial, to present patients’ perspectives and play a full part in interpretation, reporting and dissemination of findings. We follow best practise in supporting their contribution so they collaborate as equal members of the study team throughout [46, 47].

## Discussion

Given the nature of the intervention, it is not possible to blind paramedics or participants to the treatment they receive; sham FICB would be unethical. To reduce the risk of bias in reporting and analysing pain scores, paramedics record participants’ baseline pain scores before randomisation. The entire research team except the data manager will remain blind to participants’ allocations until the Trial Steering Committee has approved the primary analysis.

Variable paramedic compliance with the protocol is a danger. Hence, we shall monitor the following: use of the randomisation scratch cards to prevent subversion; missed recruitment of eligible participants; and compliance with study allocation to prevent contamination.

As we randomise participants before they consent, there is potential for biassed groups if more participants consent to follow up in one arm of the feasibility study than the other. Though we do not expect such an imbalance, the data we collect will allow us to assess this risk.

As this feasibility trial has requested volunteers to take part, they are not necessarily a representative sample of all the paramedics in the area; we acknowledge that this is a limitation. If we recommend a fully powered trial of paramedic-provided FICB, we shall invite all paramedics in the study areas to take part.

We shall compare outcomes between groups only to ensure that there are no large differences between groups and that we remain in equipoise about the clinical effectiveness of prehospital paramedic-administered FICB for hip fracture. We do not aim to evaluate clinical effectiveness in this feasibility study. Hence, we will interpret observed differences in outcomes between groups with caution in this underpowered feasibility study.

The findings of this feasibility study will enable us to recommend whether to conduct a definitive RCT of FICB by paramedics for hip fracture. If so, we shall prepare for a fully powered multi-centre trial, notably by liaising with other ambulance services and EDs to engage further sites; and drafting a proposal for research funding to evaluate whether FICB is clinically effective for patients and cost-effective for the NHS.

## Trial status

Ongoing: participant recruitment has commenced.

## Abbreviations

AE: Adverse event
ED: Emergency department
FICB: Fascia iliaca compartment block
IV: Intravenous
NHS: National Health Service
RCT: Randomised controlled trial
WAST: Welsh Ambulance Services NHS Trust
